# MRI profiling of focal cortical dysplasia using multi‐compartment diffusion models

**DOI:** 10.1111/epi.16451

**Published:** 2020-02-17

**Authors:** Sara Lorio, Sophie Adler, Roxana Gunny, Felice D’Arco, Enrico Kaden, Konrad Wagstyl, Thomas S. Jacques, Chris A. Clark, Judith Helen Cross, Torsten Baldeweg, David W. Carmichael

**Affiliations:** ^1^ Developmental Neurosciences Great Ormond Street Institute of Child Health University College London London UK; ^2^ School of Biomedical Engineering & Imaging Sciences St Thomas’ Hospital King’s College London London UK; ^3^ Great Ormond Street Hospital London UK; ^4^ Centre for Medical Image Computing University College London London UK; ^5^ Brain Mapping Unit Institute of Psychiatry University of Cambridge Cambridge UK; ^6^ Developmental Biology and Cancer Programme UCL Great Ormond Street Institute of Child Health University College London London UK; ^7^ Department of Histopathology Great Ormond Street Hospital for Children NHS Foundation Trust London UK

**Keywords:** cortical dysplasia, epileptogenic zone, multi‐compartment diffusion models

## Abstract

**Objective:**

Focal cortical dysplasia (FCD) lesion detection and subtyping remain challenging on conventional MRI. New diffusion models such as the spherical mean technique (SMT) and neurite orientation dispersion and density imaging (NODDI) provide measurements that potentially produce more specific maps of abnormal tissue microstructure. This study aims to assess the SMT and NODDI maps for computational and radiological lesion characterization compared to standard fractional anisotropy (FA) and mean diffusivity (MD).

**Methods:**

SMT, NODDI, FA, and MD maps were calculated for 33 pediatric patients with suspected FCD (18 histologically confirmed). Two neuroradiologists scored lesion visibility on clinical images and diffusion maps. Signal profile changes within lesions and homologous regions were quantified using a surface‐based approach. Diffusion parameter changes at multiple cortical depths were statistically compared between FCD type IIa and type IIb.

**Results:**

Compared to fluid‐attenuated inversion recovery (FLAIR) or T1‐weighted imaging, lesions conspicuity on NODDI intracellular volume fraction (ICVF) maps was better/equal/worse in 5/14/14 patients, respectively, while on SMT intra‐neurite volume fraction (INVF) in 3/3/27. Compared to FA or MD, lesion conspicuity on the ICVF was better/equal/worse in 27/4/2, while on the INVF in 20/7/6. Quantitative signal profiling demonstrated significant ICVF and INVF reductions in the lesions, whereas SMT microscopic mean, radial, and axial diffusivities were significantly increased. FCD type IIb exhibited greater changes than FCD type IIa. No changes were detected on FA or MD profiles.

**Significance:**

FCD lesion‐specific signal changes were found in ICVF and INVF but not in FA and MD maps. ICVF and INVF showed greater contrast than FLAIR in some cases and had consistent signal changes specific to FCD, suggesting that they could improve current presurgical pediatric epilepsy imaging protocols and can provide features useful for automated lesion detection.

**Key Points**

- We tested new advanced diffusion magnetic resonance imaging (MRI) models for the characterization of focal cortical dysplasia (FCD) lesions.
- New diffusion maps of the intracellular volume fraction and per‐axon mean, radial, and axial diffusivity are more specific to lesion changes than fractional anisotropy (FA) and mean diffusivity (MD).
- The intracellular volume fraction and per‐axon mean, radial diffusivity maps show different changes across FCD histological subtypes in 18 patients.
- These new diffusion maps have the potential to improve presurgical epilepsy MRI protocols by enhancing the characterization of FCD lesions.

## 1 INTRODUCTION

Focal cortical dysplasia (FCD) is a malformation of cortical development, and the most common cause of drug‐resistant focal epilepsy in children.^1^, ^2^ It is characterized by disrupted tissue organization with the presence of abnormal cells such as dysmorphic neurons and balloon cells.^1^ FCD lesion detection, extent identification, and microstructure characterization on magnetic resonance imaging (MRI) are crucial for planning surgical treatment^3^, ^4^; however, the radiological assessment remains challenging.^5^

Diffusion MRI can probe tissue microstructure noninvasively by measuring the diffusion process of water molecules. The most commonly used metrics are based on diffusion tensor imaging (DTI) maps such as fractional anisotropy (FA) and mean diffusivity (MD). Previous studies reported reduced FA^6^, ^7^, ^8^, ^9^ and increased MD^6^, ^9^ values in white matter regions subjacent to MRI‐visible FCD lesions. However, alterations in FA and MD distant from the FCD have also been reported,^6^, ^10^ and thus the general consensus is that these features are not specific for lesion classification.^6^, ^11^

The DTI‐based metrics FA and MD cannot differentiate between the contributions to signal changes of fiber density/orientation dispersion and diffusion across intracellular and extracellular compartments.^12^, ^13^, ^14^, ^15^ This lack of specificity hampers the neurobiological interpretation and is a confounder in the identification of pathophysiological phenomena in FCD^6^, ^15^, ^16^, ^17^ because similar signal variation can result from pathological changes or normal white matter structure.^18^

New diffusion models such as neurite orientation dispersion and density imaging (NODDI)^12^ and spherical mean technique (SMT)^13^, ^14^ account for orientation dispersion and fiber crossings within different tissue compartments, with the potential to be more specific to microstructural changes in FCD lesions.^15^ These multi‐compartment models require a greater range of diffusion data, which until recently would have required clinically impractical scan times, particularly for pediatric patients.

A preliminary study used NODDI in five patients with suspected FCD,^15^ showing that the intracellular volume fraction (ICVF) map enhanced lesion contrast.^15^ The SMT multi‐compartment microscopic diffusion was used to detect an altered intracellular and/or extracellular neuropathological process in mouse brains affected by tuberous sclerosis complex,^13^ the brain lesions of which share histopathological features with FCD type IIb.^19^

In this study, we aimed to determine whether the diffusion parameters from multi‐compartment models demonstrated consistent changes in suspected FCD lesions, to determine whether they were more sensitive than FA and MD, and to test their ability to characterize tissue property differences between histological subtypes. To this end, we used recent advances in MRI software and hardware to obtain data suitable for SMT and NODDI techniques in ~7 minutes. We investigated a retrospective cohort of more than 30 patients with suspected FCD, utilizing NODDI, microscopic diffusion tensor, and multi‐compartment microscopic diffusion SMT. All patients had MRI‐positive lesions on clinical three‐dimensional (3D) fluid‐attenuated inversion recovery (FLAIR) or T1‐weighted images.

First, two neuroradiologists visually assessed the lesion conspicuity on the new diffusion parameters, FA, MD maps and optimised epilepsy protocol images^20^ (3D T1‐weighted and 3D‐FLAIR) to compare lesion contrast on different MR images. Second, changes in diffusion parameters were quantified using profiles across different cortical and subcortical depths and compared with homotopic healthy regions. Finally, we assessed if the new diffusion map profiles were specific to FCD histological subtypes.

## 2 METHODS

### 2.1 Participants

A retrospective cohort of 33 pediatric patients (mean age ± SD = 10 ± 4 years, range = 2‐21 years, female = 15) was identified for this research study from all those undergoing assessment for epilepsy surgery at Great Ormond Street Hospital (GOSH), following approval by the national research ethics service. The inclusion criteria were patients with radiological and electroclinical diagnosis compatible with FCD and patients who had 3T MRI at GOSH with the full epilepsy imaging protocol that included multi‐shell diffusion. Patients younger than 2 years of age with MRI scans showing severe motion artifacts (ie, indistinguishable adjacent gyri due to motion or severe ringing), or without the full protocol described in the following section were excluded. The 33 patients included in the study represent the radiologically defined group that was used to test visually and quantitatively signal changes on the multi‐compartment diffusion maps.

### 2.2 Magnetic resonance imaging

All patients were scanned on a 3T whole‐body MRI system (Magnetom Prisma, Siemens Medical Systems), using a 20‐channel receive head coil and body coil for transmission and 80 mT/m magnetic field gradients. Three‐dimensional structural T1‐weighted (T1w) images were acquired using magnetization‐prepared rapid gradient‐echo (MPRAGE) (echo time/repetition time [TR/TE] = 2300/2.74 ms, field of view [FOV] = 256 × 256 mm^2^, flip angle = 8°, voxel size = 1 × 1 × 1 mm^3^), FLAIR (TR/TE/inversion time [TI] = 4000/395/1800 ms, FOV = 256 × 256 mm^2^, flip angle = 120°, voxel size = 0.65 × 1 × 0.65 mm^3^), and a diffusion‐weighted protocol. The multi‐direction diffusion sequence was included primarily to provide state‐of‐the‐art white matter tractography data for patients going forward to surgery without requiring further imaging. This employed a diffusion‐weighted spin‐echo single‐shot 2D echo‐planar imaging (EPI) acquisition, field of view = 220 × 220 mm^2^, matrix size = 110 × 110, in‐plane voxel resolution = 2.0 mm, GRAPPA factor 2, phase‐encoding (PE) partial Fourier = 6/8. Multiband radio frequency pulses allowed simultaneous multi‐slice acquisition.^21^, ^22^ A multiband factor of 2 was employed, halving the time required to obtain the 66 slices (2 mm thickness with 0.2 mm gap). Diffusion gradients were applied over two shells: *b* = 1000, 2200 s/mm^2^, with 60 non‐colinear diffusion directions per shell, in addition to 13 interleaved *b* = 0 (b_0_) (non–diffusion‐weighted) images. The gradient strength and eddy current performance enabled monopolar diffusion encoding with TE = 60 ms and TR = 3050 ms, thereby limiting the total acquisition time to 7 minutes 20 seconds. For the correction of magnetic susceptibility‐related distortions (see Diffusion processing), an additional single b_0_ acquisition was performed, with the PE direction flipped by 180° (in the anterior‐posterior direction); all other parameters were unchanged.

### 2.3 Diffusion processing and map estimation

We applied four different techniques to model the diffusion signal across brain tissue:

- Model 1: DTI providing FA and MD maps;
- Model 2: NODDI providing ICVF and orientation dispersion (OD) maps;
- Model 3: SMT microscopic (μ) diffusion tensor estimating μ axial diffusivity (μAD), μ radial diffusivity (μRD), μFA, and μMD maps;
- Model 4: SMT multi‐compartment microscopic diffusion computing intra‐neurite volume fraction (INVF), intrinsic diffusivity, extra‐neurite μRD and extra‐neurite μMD maps.

The preprocessing of the diffusion‐weighted data was the same for all models and was performed using FSL5.0 (http://www.fmrib.ox.ac.uk/fsl). To estimate and correct susceptibility‐induced distortions, the diffusion‐weighted data were combined with the PE‐flipped b_0_ image^23^ using the “topup” function. Then, eddy current and susceptibility distortions were removed using the “eddy” function before the brain extraction tool (BET)^24^ was applied to skull‐strip the brain volume.

The diffusion tensor model was fitted to the corrected multi‐shell data using “dtifit” with a weighted least‐squares fit, and FA and MD maps were calculated.

The NODDI ICVF and OD maps were computed for the brain voxel using the NODDI Matlab Toolbox (http://www.nitrc.org/projects/noddi_toolbox) 12 with default settings. Briefly, NODDI models diffusion in each voxel as three independent compartments: intra‐neurite, extra‐neurite and free water compartment, assuming fixed‐compartment diffusivities.^12^ The intra‐neurite compartment characterizes the space occupied by neurites and is modeled by a set of “sticks.” The extra‐neurite compartment models water diffusing in the space around neurites, while the free water compartment represents the free water diffusion (ie, cerebrospinal fluid [CSF]). The ICVF provides a measure of cell density as a fraction of the non‐CSF compartment, whereas the OD estimates the orientation distribution of the intra‐neurite compartment.

For the SMT models, the corrected diffusion‐weighted data were smoothed with an isotropic Gaussian kernel of 2 mm full width at half maximum (FWHM) to remove Gibbs artifacts. The SMT toolbox 0.3 (https://github.com/ekaden/smt) was used to estimate the microscopic diffusion tensor and multi‐compartment microscopic diffusion maps adjusted for the signal offset induced by Rician noise.^25^ The model assumed variable diffusivity across the brain, with maximum value set to 4 × 10^−3^ mm^2^/s.^13^, ^14^ The microscopic tensor maps are per‐axon effective diffusion coefficients unconfounded by the intra‐voxel fiber orientation distribution, such as µAD, µRD, and µMD. The multi‐compartment microscopic diffusion maps represent estimates of diffusion features specific to the intra‐ and extra‐neurite compartments without the confounding effects of complex fiber orientation distribution, including the INVF and intrinsic diffusivity.

#### 2.3.1 Visual assessment

To compare the lesion visibility on different MRI contrasts, two neuro‐radiologists (R.G., F.D.’A.) were presented with coregistered T1w, FLAIR, FA, MD, SMT, and NODDI images of MR lesion‐positive patients. Following standard radiological practice, they compared the different contrasts in the same patient; this allowed them to visually assess lesion location and relative conspicuity in the different image types. A lesion visibility score from 1 to 4 (1 = not visible, 2 = subtle, 3 = visible, 4 = clearly visible) was assigned to each image type for each patient. Neither of the observers were blinded to radiological or EEG reports. Intensity windowing was individually adapted to gain optimal contrast. The neuroradiologists assessed the images independently and so were blinded to each other's ratings.

To determine the level of agreement between the two raters, intraclass correlation coefficient (ICC) was estimated using a two‐way random‐effects model based on two raters and absolute agreement^26^ implemented in IBM SPSS v25. The ICC values along with 95% confidence intervals (CIs) and *P*‐values were reported for each image in Table 1. Interpretation of the ICC values was as follows: <0.50, poor agreement; between 0.50 and 0.75, moderate, between 0.75 and 0.90 good; above 0.90, excellent.^26^ The ICC values were corrected for chance of agreement.

**Table 1:** Measure of inter‐rater agreement on visual scores

Maps	ICC		
	Mean value	*P*‐value	95% confidence interval Lower − upper bound
FLAIR	0.75	<10^−5^	0.51‐0.88
MPRAGE	0.69	.001	0.38‐0.84
ICVF	0.51	.012	0.05‐0.75
ODI	0.52	.021	0.03‐0.76
μFA	0.49	.035	0.01‐0.75
μAD	0.48	.031	0.1‐0.74
μMD	0.58	.003	0.153‐0.79
μRD	0.64	.001	0.29‐0.82
Diff	0.79	<10^−5^	0.56‐0.9
μExtra‐neurite MD	0.66	.001	0.32‐0.83
μExtra‐neurite RD	0.77	<10^−5^	0.51‐0.89
INVF	0.67	.001	0.33‐0.83
FA	0.26	.129	‐
MD	0.45	.007	0.01‐0.73

Note

Two expert neuroradiologists scored the FCD lesion visibility on FLAIR, MPRAGE, NODDI, microscopic and multi‐compartment microscopic SMT, and standard DTI images. Inter‐rater agreement was assessed using intra‐class coefficient (ICC), corrected for chance of agreement. For each image, we report the index value, the *P*‐value, and the confidence interval for *α* = 95%. Significance level was set at *P*‐value < .05.

Abbreviations: Diff, intrinsic diffusivity; ICVF, intracellular volume fraction; INVF, intra‐neurite volume fraction; ODI, orientation dispersion index; μAD, microscopic axial diffusivity; μExtra‐neurite MD, extra‐neurite microscopic mean diffusivity; μExtra‐neurite RD, extra‐neurite microscopic radial diffusivity; μFA, microscopic fractional anisotropy; μMD, μ mean diffusivity; μRD, microscopic radial diffusivity.

Aiming to compare lesion conspicuity scores between T1w, FLAIR, and diffusion images across patients, we applied the Friedman test to the mean score of the two radiologists computed for each map. Then, we performed a multiple comparison test between the ranking means provided by the Friedman test for each group of images. We set statistical significance at *P* < .05 after applying the Bonferroni correction for multiple comparisons.

### 2.4 Quantitative cortical and subcortical sampling

**Figure 1:**
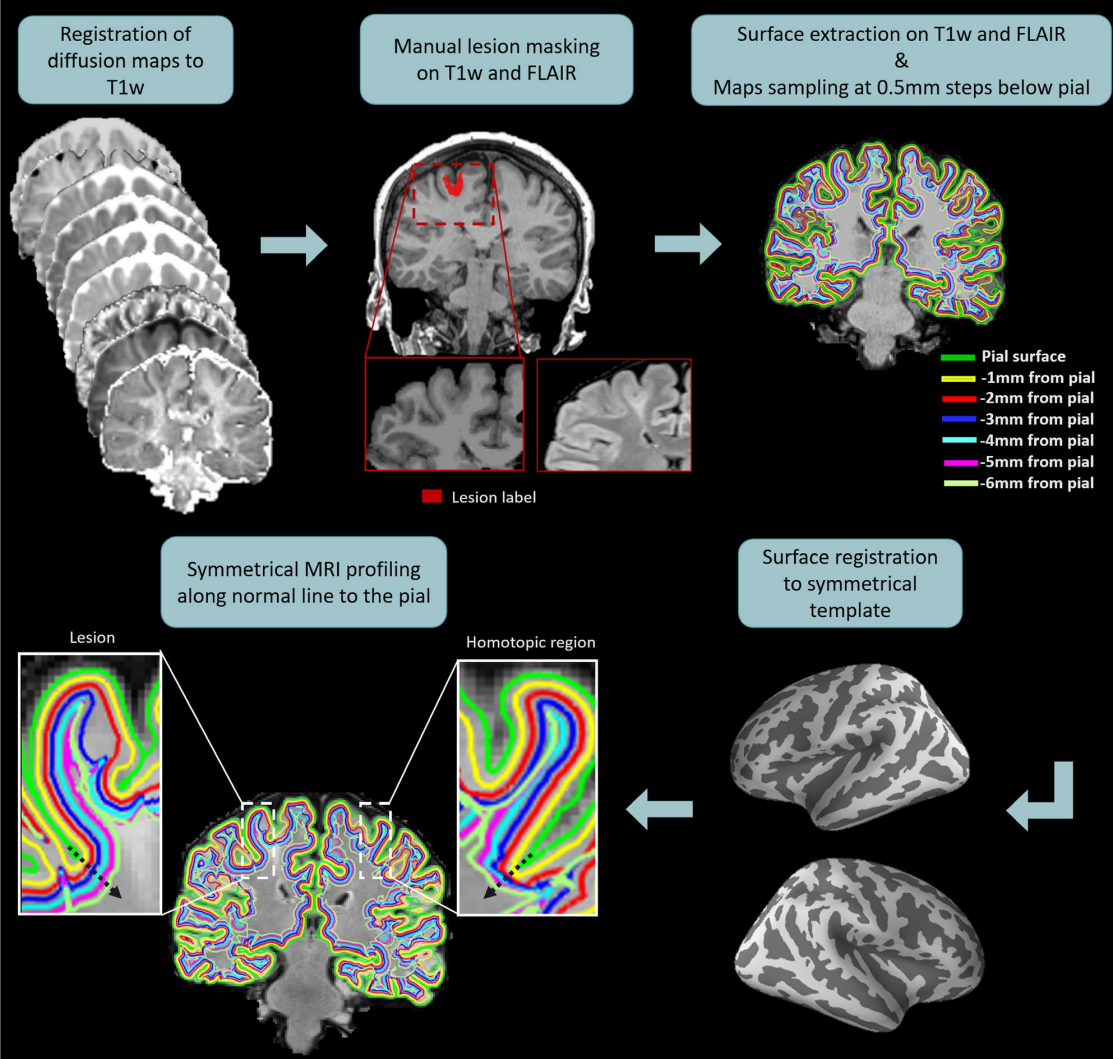
Maps sampling workflow. First, the new diffusion maps were coregistered to the T1‐weighted (T1w) data. Manual lesion masking and surface extraction at increasing depths were performed on T1w and fluid‐attenuated inversion recovery (FLAIR) images. The diffusion maps were projected onto the surfaces and sampled from the pial surface. Then, the surface sampling and the lesion masks were spatially registered to a symmetric template, allowing the symmetrical MRI profiling of the diffusion maps along the normal (black arrow) to the pial surface for the lesion and the homotopic region

Figure 1 shows the workflow for the sampling of the diffusion data. FreeSurfer software v5.3^27^ was used to co‐register the diffusion maps and FLAIR to the T1w, and to reconstruct cortical‐subcortical surfaces. Both FLAIR and T1w images were employed to generate the accurate smooth mesh representations of the pial surface.^27^, ^28^

To examine the intracortical and subcortical signal of the diffusion maps, we sampled the diffusion values from the pial surface at steps of 0.5 mm down to 6 mm, guided by a straight line providing vertex correspondence across surfaces.^29^ We note that the gray matter/white matter (GM/WM) border was not used because of the tendency for lesions to cause tissue misclassification owing to its defining feature being a loss of GM/WM differentiation. The diffusion parameter map surfaces (sampled at different depths) were smoothed using a 10 mm FWHM Gaussian kernel. Finally, we registered the sampled diffusion maps to an average symmetric space having an identical number of vertices for each hemisphere.^30^ This allowed us to analyze diffusion changes between homologous regions and therefore control for differences in cortical thickness due to anatomical variability by using an anatomically matched internal control.

### 2.5 Lesion masks

FCD lesions were identified on T1w and FLAIR images by an experienced pediatric neuroradiologist. 3D binary masks were delineated manually for the 33 patients. The lesion masks were first registered onto the surface reconstructions and then to the symmetric template. This procedure provided a mask for the lesion and one for the homologous healthy tissue.

### 2.6 Statistical analysis

#### 2.6.1 Lesion profiling

MRI profiles for each diffusion map were obtained by averaging the values within the patient's lesion mask and homologous region, separately, along each sampling surface. To investigate signal changes in FCD lesions, the profiles of the diffusion maps were statistically compared between lesion and homologous region for the radiologically defined groups (33 patients).

We used a paired *t* test to evaluate diffusion changes within each parameter map at various sampling depths. Correction for multiple comparisons was applied using false discovery rate (FDR) at *P* < .05. The data normality, required for the *t* test, was assessed using the Shapiro‐Wilk test run on the MRI profiles of the lesions and homologous regions.

#### 2.6.2 Histological subtype profiling

To quantitatively study pathology‐specific changes in the diffusion maps, we repeated the *lesion profiling* analysis described above in the subgroup of patients with histologically confirmed FCD lesions (4 FCD type IIa and 14 FCD type IIb). The diffusion maps that showed a significant difference across cortical depth in the histologically defined group were employed to estimate the asymmetry of the MRI profiling by computing the diffusion values difference between the lesion and homologous region at each sampling depth.

The MRI profiling asymmetry measures were compared between patients with histologically confirmed FCD type IIa and type IIb using a two‐sample *t* test. Correction for multiple comparisons was applied using FDR at *q* < 0.05.

#### 2.6.3 Correlation between visual scores and lesion profiling

The correlation between the mean visual score of the two radiologists computed for each diffusion map, and the asymmetry of the MRI profiling was estimated using the Spearman correlation coefficient as described in the Appendix S1. This was performed in the radiologically defined group (n = 33).

## 3 RESULTS

### 3.1 Patients’ clinical information

The patients’ demographics and clinical information can be found in Table S1. A total of 25/33 patients underwent surgery: 18 were histologically diagnosed as FCD (14 FCD type IIb, 4 FCD type IIa), 1 had minimally invasive surgery with thermal ablation and hence no histology is available, 1 had polymicrogyria, 2 had glioneuronal tumours, and 3 were diagnosed with hippocampal sclerosis (2 had standard temporal lobe resection involving the anterior temporal lobe, and 1 had resection also in the cingulate, which did not exhibit any histopathological abnormality). Seizure freedom was achieved in 21/25 cases at 1.5 years after surgery.

### 3.2 Visual assessment

**Figure 2:**
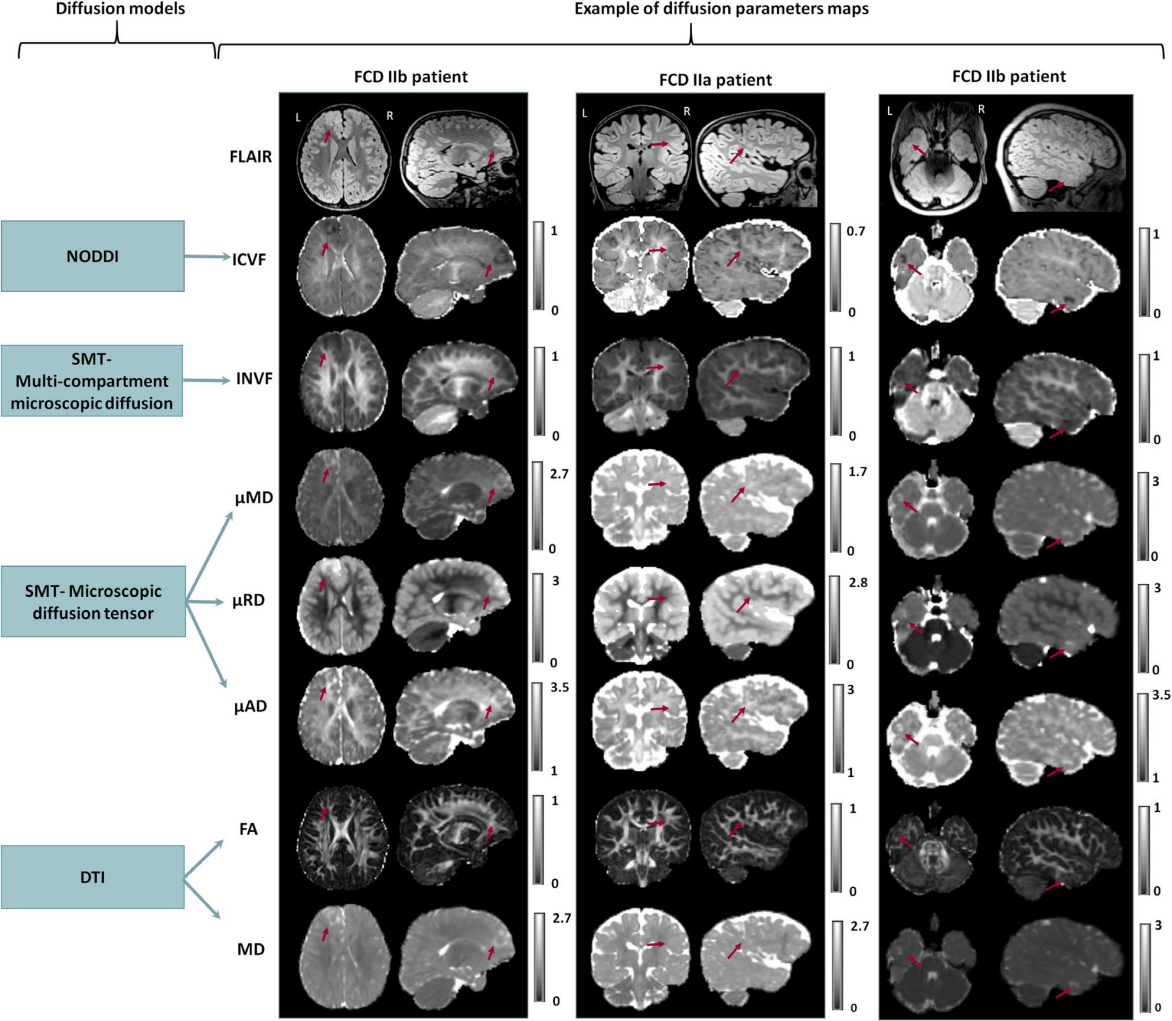
Diffusion models and examples of diffusion parameters maps were obtained in a clinical setting. Example of FLAIR, neurite orientation dispersion and density imaging (NODDI) intracellular volume fraction (ICVF), spherical mean technique (SMT) multi‐compartment microscopic intra‐neurite volume fraction (INVF), SMT microscopic mean diffusivity (μMD), radial and axial diffusivities (µRD and µAD), and standard DTI fractional anisotropy (FA) and mean diffusivity (MD) maps for three patients. (left) FCD IIb patient with very well‐delineated lesion on all images except FA. (center) FCD IIa patient showing lesion is poorly delineated by FLAIR, FA, and MD but visible on ICVF and μRD maps. (right) FCD IIb patient showing the lesion is poorly delineated by FLAIR, FA, MD, but very well visible on ICVF, μMD, μRD, and μAD maps

Figure 2 shows examples of FCD type IIa and type IIb lesions clearly visible on ICVF, INVF, μMD, μRD, and μAD maps. Compared to the best visualization achieved in either FLAIR or T1w, the lesion conspicuity was visually assessed as being better/equal/worse: on the ICVF in, respectively, 5/14/14 individuals, on the INVF in 3/3/27, on the μRD in 3/1/29, on the μMD in 2/3/28, and on the μAD in 1/1/31 patients. Similar to the ICVF map, the lesion conspicuity on the T1w was better/equal/worse in 5/11/17 cases compared to FLAIR.

Compared to the best visualization achieved between FA and MD maps, the lesion conspicuity was visually assessed as being better/equal/worse on the ICVF maps, respectively, in 27/4/2 cases, on the INVF in 20/7/6, on the µMD in 10/12/11, on the μRD in 17/10/6, and on the μAD in 11/10/12 patients.

The mean scores of lesion conspicuity for each image type and patient can be found in Table S3.

The ICC index showed a significant (*P* < .05) moderate agreement (0.5 < ICC < 0.75) for the clinical FLAIR MPRAGE, ICVF, and the majority of diffusion maps, as shown in Table 1. The µFA, µAD, FA, and MD exhibited poor agreement (ICC < 0.5), as reported in Table 1.

**Figure 3:**
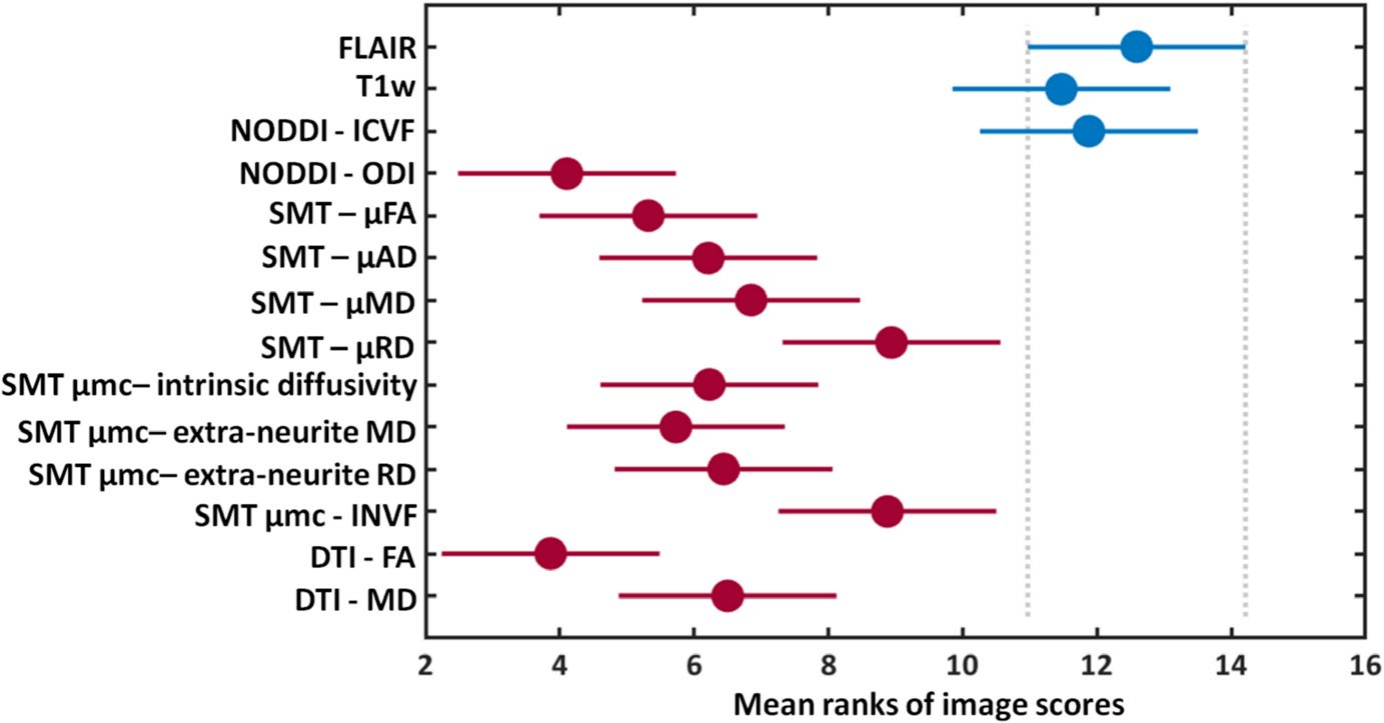
Multiple comparison test on the mean ranks of the image scores. The graph shows that the T1w and NODDI intracellular volume fraction (ICVF) have mean ranks not significantly different (blue) from FLAIR, whereas the other diffusion maps have mean ranks significantly different (red) from FLAIR images. Circles represent mean rank values, and error bars correspond to standard deviations. Extra‐neurite MD, extra‐neurite microscopic mean diffusivity; extra‐neurite RD, extra‐neurite microscopic radial diffusivity; INVF, intra‐neurite volume fraction; ODI, orientation dispersion index; μAD, microscopic axial diffusivity; μFA, microscopic fractional anisotropy; μMD, microscopic mean diffusivity; μRD, microscopic radial diffusivity

There was a statistically significant difference in lesion conspicuity scoring across image types (*P* < .001). The post hoc multiple comparison test applied to the Friedman ranking test showed that the mean ranking of FLAIR, T1w, and ICVF maps was not significantly different, whereas the mean ranking of FA, MD, other NODDI maps, multi‐compartment microscopic, and microscopic SMT was significantly reduced compared to FLAIR images (*P* < .05; see Figure 3).

### 3.3 Quantitative lesion profiling

**Figure 4:**
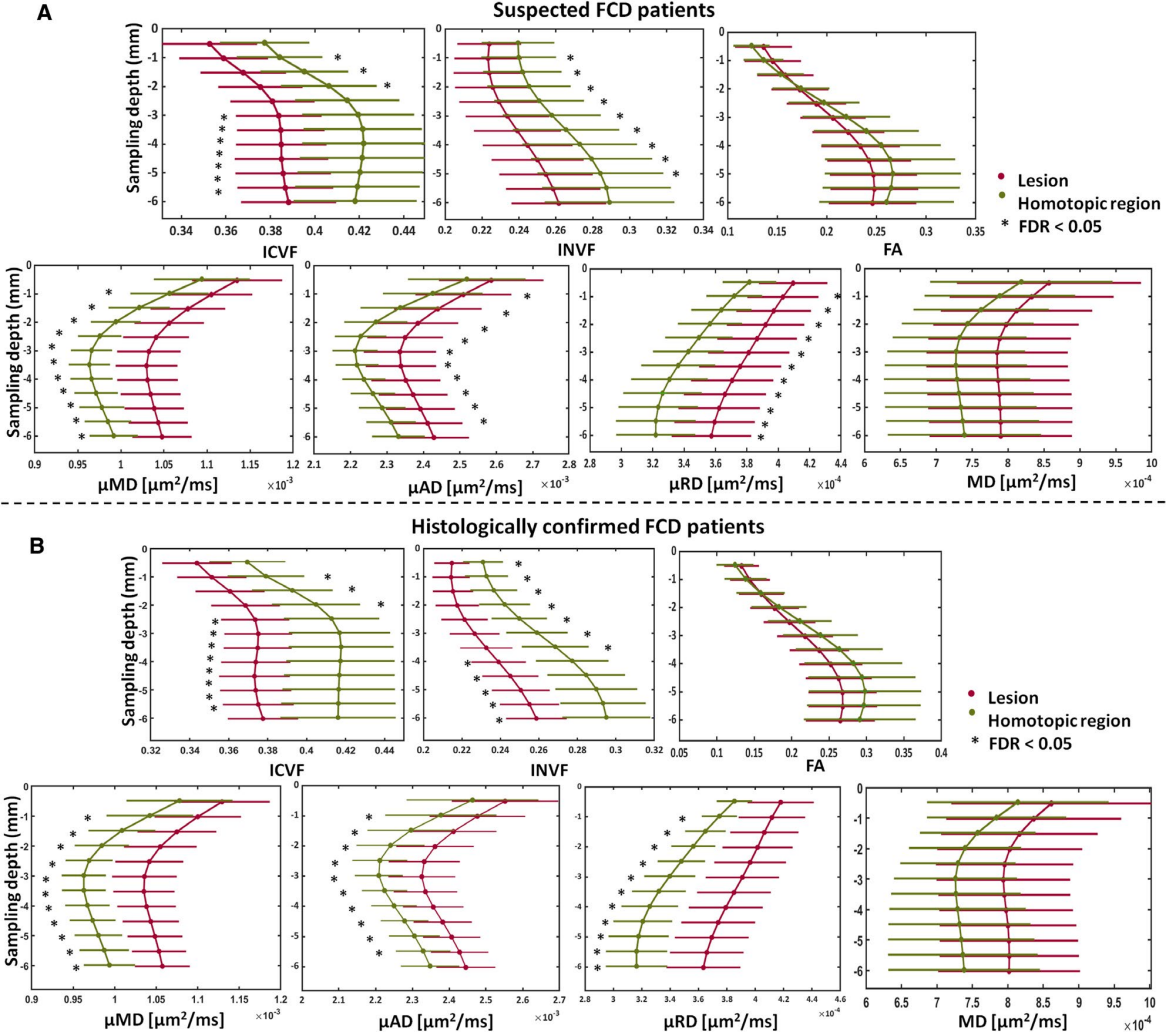
MRI profiling of FCD lesions and homotopic regions. A, MRI profiling of FCD lesions (red) and homotopic regions (green) for the new diffusion maps showing significant profile changes at false discovery rate (FDR) <0.05 (*), and for MD, FA maps not providing significant differences for the whole patient cohort. The sampling depth is reported as a distance from the pial surface (0 mm). B, MRI profiling of FCD lesions (red) and homotopic (green) regions on histologically confirmed patients showing significant (false discovery rate [FDR] <0.05) profile changes for all sampling depths on new diffusion maps in contrast to MD, FA maps. FA, fractional anisotropy; ICVF, intracellular volume fraction; INVF, intra‐neurite volume fraction; MD, mean diffusivity; μAD, μ axial diffusivity; μMD, μ mean diffusivity; μRD, μ radial diffusivity. Error bars correspond to standard deviations computed over the cohort of patients

We observed significant (*P*_FDR_ < .05) diffusion value changes between the suspected FCD lesions and their homologous regions on the ICVF, INVF, μMD, μAD, and μRD maps for the entire radiologically defined cohort (see Figure 4A). Significant signal reduction was found within the lesions on the ICVF and IVNF maps at 2‐5.5 mm depth for both groups (see Figure 4A). The μMD, μAD, and μRD were significantly increased in the lesion at 2‐5 mm cortical depth, with respect to the healthy homologous regions for both groups, as shown in Figure 4A.

No significant differences were observed for the other NODDI and SMT maps or on the DTI FA and MD images.

### 3.4 Histological subtype profiling

The lesion profiling analysis performed on the histologically confirmed group showed results that were similar to the ones obtained for the radiologically defined group. Significant signal reduction was found within the lesions on the ICVF and IVNF maps at 2‐5.5 mm depth, while the μMD, μAD, and μRD were significantly increased in the lesion with respect to the healthy homologous regions (see Figure 4B).

**Figure 5:**
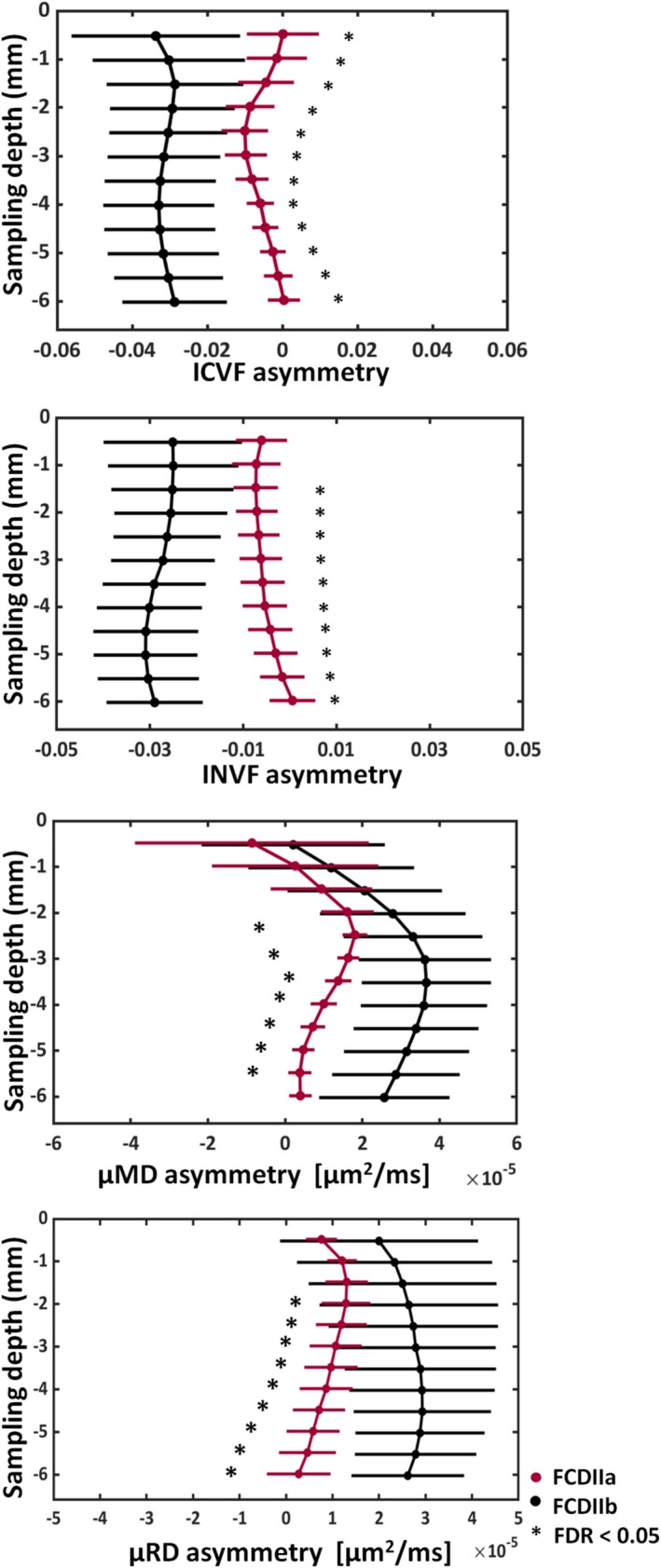
MRI profiling of the asymmetry for FCD IIa and IIb patients. Significant asymmetry differences at false discovery rate (FDR) <0.05 (*), between histologically confirmed FCD IIb (black) and FCD IIa (red) patients were found on the ICVF, INVF, μMD, and μRD. The asymmetry was estimated by computing the difference vertex‐wise between the lesion and homotopic region values using the maps showing significant changes between the two regions. The sampling depth is reported as a distance from the pial surface (0 mm). ICVF, intracellular volume fraction; INVF, intra‐neurite volume fraction; μMD, μ mean diffusivity; μRD, μRadial diffusivity; μAD, μAxial diffusivity. Error bars correspond to standard deviations computed over the cohort of patients

The asymmetry profile analysis performed on those maps showing significant changes in lesion for both the radiologically and histologically confirmed groups, demonstrated that FCD type IIb lesions had significant (*P*_FDR_ < 0.05) increased asymmetry with respect to FCD type IIa on the μMD and μRD (Figure 5). The asymmetry profiling changes in FCD type IIb involved all sampling depths. The asymmetry measures for FCD type IIa showed subtle signal alterations at 1.5‐4 mm depth on the μRD, and at 1.5‐3 mm depth on the μMD.

FCD type IIb demonstrated significant asymmetry changes with respect to FCD type IIa on the ICVF and INVF maps (Figure 5). The asymmetry profiles showed ICVF and INVF reduction in type IIb lesions with respect to homologous regions involving all sampling depths, while type IIa exhibited subtle ICVF asymmetry mainly located at 1.5‐3mm depth and absence of signal changes on the INVF maps. No significant asymmetry differences between subtypes were found for the µAD.

Because FA and MD did not show significant changes between the lesion and the healthy region in the histologically confirmed patients, those maps were not included in the histological subtype profiling analysis.

### 3.5 Correlation between visual scores and lesion profiling

µRD and μExtra‐neurite RD maps had the highest correlation between visual scores and lesion profiling (respectively, *ρ* = 0.59, 0.56 *P*‐value < .01), whereas ICVF, INVF, µFA, FA, and MD had correlation smaller than 0.5 at *P*‐value < .05 (see Table S3).

## 4 DISCUSSION

To our knowledge, this is the first study evaluating the ability of multi‐compartment diffusion maps based on SMT and NODDI models, to delineate and characterize suspected FCD lesions in a significant pediatric population with drug‐resistant focal epilepsy. This was made possible by recent advances in MRI hardware, such as improved scanner gradient performance, and software, such as multiband imaging sequences^21^, ^22^ that reduce the acquisition time of multi‐shell diffusion data (~7 minutes) allowing for its incorporation into a clinical pediatric epilepsy protocol.

### 4.1 Visual assessment

Based on the multiple comparison statistical tests, the ICVF map provided comparable FCD lesion conspicuity to FLAIR and T1w images and improved it with respect to MD and FA maps. At the individual level, the lesion contrast on ICVF was enhanced in ~10% of patients; therefore, this map is most likely to provide information in combination with FLAIR to detect and demarcate FCD. Radiological evaluation is strengthened by observing signal abnormalities in several image modalities^20^; therefore, it is beneficial to combine the contrast of these new diffusion maps with FLAIR and T1‐weighted images. As in many patients, conventional imaging based on visual evaluation is unable to pinpoint the epileptogenic lesion,^31^, ^32^ it is crucial to test if these new image contrasts may increase detection rates,^33^ particularly in MRI‐negative patients.

The moderate agreement observed between the qualitative scoring of the two raters is concordant with previous literature studies reporting similar results for T2‐weighted, FLAIR, and T1w.^34^ Differing assessments of the diffusion maps could be explained by the relative lack of experience in viewing these contrasts and lower resolution of the diffusion maps (approximately half) compared to FLAIR and T1w images.

### 4.2 Quantitative lesion profiling

In agreement with the visual analysis, quantitative investigation of ICVF, and additionally INVF, μMD, μRD, and μAD parameters showed significant alterations in suspected and histologically confirmed FCD lesions at different sampling depths. In agreement with the previous study of five adult patients with suspected FCD,^15^ we observed decreased ICVF signal in the lesions. Similarly, we found significant INVF decrease in suspected and confirmed FCD lesions. Both ICVF and INVF have been proposed as biomarkers of highly anisotropic structures, such as neurons nuclei and glia, as suggested by previous studies on multiple sclerosis,^35^ Alzheimer disease,^36^ and Parkinson disease.^37^ The within‐lesion decreases are concordant with altered extracellular diffusion and increased extra‐neurite volume measures performed on histology samples from surgical resections.^38^ Those phenomena could also reflect the formation of additional diffusion barriers that may arise from loss of cortical stratification, deposits of extracellular matrix molecules, or morphological changes of astrocytic processes, usually associated with tissue remodeling due to astrogliosis.^38^

However, there are differences in the estimation of these two maps. In contrast to NODDI ICVF, the SMT multi‐compartment INVF estimates the intrinsic diffusivity from the data instead of assuming it to be equal to a fixed value.^12^, ^13^ The differences in the number of cases where the ICVF improved lesion conspicuity with respect to the INVF might also be explained by the fact that the diffusion data were smoothed before estimating the SMT maps to reduce their sensitivity to Gibbs ringing artifacts. The smoothing could enhance potential partial volume effects between subjacent structures.

The increase in both μRD and μAD is in agreement with an increase of μMD and explains the lack of signal changes on the μFA maps. Because μRD and μAD parameters are thought to be more specific to key features of brain microanatomy, they might be able to identify signal changes underlying a defect of neurogenesis or the presence of altered cells such as dysmorphic neurons and balloon cells.^13^, ^14^ As malformations of cortical development affect the tissue microstructure underlying cortical layers and white matter tracts,^38^, ^39^ the NODDI and SMT maps could now better characterize the presence of decreased myelinated axons and neurites resulting in increased extracellular space,^6^ or the presence of abnormal cells.

In contrast to some other studies, we did not observe any significant changes on FA and MD maps for either the radiological or the histologically confirmed FCD lesions. This might be explained by the fact that those DTI maps are affected by healthy variability in underlying tissue properties including neuronal density, fiber orientation dispersion, axonal diameter, and degree of myelination, and ignore the presence of multiple tissue components, which can hinder signal changes induced by pathological phenomena in our sample size.

In this study, we were interested primarily in characterizing the presence of consistent changes in quantitative diffusion maps via MRI profiling. Due to their ability to probe tissue biophysical properties in vivo*,* they have the potential to bridge the gap between radiological assessment and ex vivo histology.^17^ For this reason, we did not apply the profiling analysis to FLAIR and T1w images, which have already been characterized.^16^ Although these clinical images provide good contrast in some FCD lesions, the intensity is not quantitative, thereby limiting their specificity to microstructural tissue properties.^40^

### 4.3 Histological subtype profiling/neurobiological interpretation

Moreover, we showed that multi‐compartment diffusion maps could help the characterization of subtypes, as FCD IIb lesions exhibited enhanced signal changes on the ICVF, INVF, μMD, and μRD maps compared to FCD IIa, where signal alterations were subtle and affected layers closer to the pial surface. The presence of balloon cells in deep cortical and subcortical layers in FCD IIb might explain the altered cellular density measured by ICVF and INVF maps and the disrupted diffusivity quantified by μMD and μRD maps. These results are in agreement with those of previous studies analyzing cellular water diffusivity on histological samples.^38^ Because our study has been carried out on 4 FCD IIa and 14 FCD IIb lesions, further investigations on larger patient populations are necessary to validate the ability of multi‐compartment diffusion images to capture histopathology subtype. Nevertheless, the consistent signal changes we have demonstrated in modest numbers (despite being the largest study of its type) strongly motivate further evaluation.

### 4.4 Limitations and outlook

Because the clinical trend is to perform surgical resection on younger patients with clearly defined epileptogenic regions and seizures that affect both quality of life and development, it is crucial to noninvasively characterize FCD lesions for surgical planning, seizure freedom, and neurodevelopmental outcome.^17^, ^41^

The consistency of changes in ICVF, μMD, μRD and μAD, and INVF for both the radiologically defined lesions and the subset with histological confirmation indicates that these maps could be useful for the visual identification of lesions and the integration into algorithms for automated detection.^42^ Furthermore, some evidence of changes specific to FCD subtype was found that may indicate that these measures have the potential to reveal underlying tissue properties in FCD lesions noninvasively. Future studies are needed to validate these maps in larger cohorts including in MRI‐negative patients.

In this work, NODDI and SMT techniques were applied to investigate changes in both white and cortical gray matter. Those models rely on strong assumptions; if the tissue properties significantly differ from those constraints, the model can lead to erroneous interpretations, as might be the case in cortical gray matter.^43^ Despite criticisms raised regarding the model assumptions,^43^ NODDI is frequently used to study the human cortex.^44^, ^45^, ^46^

In conclusion, we have demonstrated that the multi‐compartment diffusion maps showed changes in FCD lesions compatible with underlying disrupted tissue microstructure and could be valuable features for characterizing the affected area and identifying the histological subtypes.

## 5 STUDY FUNDING AND ACKNOWLEDGEMENT

This research was funded by the Henry Smith Charity and Action Medical Research (GN2214). This research was supported by the National Institute of Health Research (NIHR) Great Ormond Street Hospital Biomedical Research Centre. The views expressed are those of the author(s) and not necessarily those of the National Health Service, the NIHR or the Department of Health. D.C. and S.L. are supported by the King's College London Wellcome/EPSRC Centre for Medical Engineering (WT 203148/Z/16/Z). S.A. received funding from the Rosetrees Trust. T.S.J. receives funding from Great Ormond Street Children's Charity, The Brain Tumour Charity, Children with Cancer UK, Cancer Research UK, and the Olivia Hodson Cancer Fund.

We would like to thank the Centre for Magnetic Resonance Research at the University of Minnesota for providing the multiband‐EPI sequence (http://www.cmrr.umn.edu/multiband) used in this work.

## CONFLICT OF INTEREST

None of the authors has any conflict of interest to disclose. We confirm that we have read the Journal's position on issues involved in ethical publication and affirm that this report is consistent with those guidelines.
