# Supplementary material for: MRI profiling of focal cortical dysplasia using multi‐compartment diffusion models
Source: Epilepsia. 2020 Feb 17;61(3):433–44. doi: 10.1111/epi.16451 (PMC7154549; doi:10.1111/epi.16451)
Supplement: Supplementary file 1 [file EPI-61-433-s001.docx]

**Supplementary material**

Correlation between visual scores and lesion profiling

We estimated the correlation between the mean visual score of the two radiologists computed for each diffusion map, and the asymmetry of the MRI profiling using the Spearman correlation coefficient. The asymmetry was computed by subtracting the diffusion values of the homologous healthy regions from the ones within the lesion at 3mm sampling from the pial surface. The 3mm sampling procedure was utilized to be conservatively close to the grey/white border that is usually characterised by abnormal intensity changes in FCD lesions

| Patient | Sex | | Age (y) | Disease onset | Disease duration (y) | MRI | | | Histology | Surgery | Engel |
| --- | --- | --- | --- | --- | --- | --- | --- | --- | --- | --- | --- |
|  |  |  |  |  |  | **Lesion site** | **Hemisphere** | **Radiological report** |  |  |  |
| 1 | m | 3 | | 22m | 1.93 | par-occ | R | focal signal abnormality | Glioneuronal tumour | y | Ia |
| 2 | f | 5 | | 3y | 2.10 | middle fr gyrus | L | focal thickening, blurring | FCD IIb | y | Ia |
| 3 | f | 15 | | 2y | 13 | pre-fr gyrus, sup fr sulcus | R | transmantle sign | FCD IIb | y | Ia |
| 4 | m | 16.00 | | 3y | 13.00 | fr gyrus, fr operculum | R | transmantle sign | FCD IIb | y | Ia |
| 5 | f | 8 | | 28m | 6.21 | par operculum | L | abnormal cortical folding, blurring | FCD IIb | y | IV |
| 6 | m | 4 | | 3y | 1 | post fr lobe | R | transmantle sign | FCD IIa | y | Ia |
| 7 | m | 15 | | 2m | 15 | paracentral lobule | L | transmantle sign | FCD IIb | y | Ia |
| 8 | f | 16 | | 6y | 10 | fr gyrus | L | transmantle sign | Not resected | n | Ia |
|  |  |  |  |  |  | temp pole |  | hippocampal sclerosis | HS (ILAE 3) | y |  |
| 9 | m | 9 | | 3d | 9 | fr | R | extensive malformation of cortical development | FCD IIa | y | III |
| 10 | m | 8 | | 4y | 4 | post temp lobe | R | transmantle sign | NA | n |  |
| 11 | f | 17 | | 1y | 16 | cingulate gyrus | L | focal signal abnormality | No abnormality | y | Ia |
|  |  |  |  |  |  | mesial temp |  | hippocampal sclerosis | HS (ILAE 1) | y |  |
| 12 | f | 7 | | 9m | 6.68 | fr, temp, par | R | focal signal abnormality | NA | n |  |
| 13 | m | 18 | | 1y | 17 | inferior temp sulcus | L | transmantle sign | FCD IIb | y | Ia |
| 14 | f | 11 | | 9y | 3 | par | R | atrophy | NA | n |  |
|  |  |  |  |  |  | temp | L | white matter signal change |  |  |  |
| 15 | m | 11 | | 2y | 9 | superior temp gyrus | L | transmantle sign | FCD IIb | y | Ia |
| 16 | m | 2 | | 10w | 2 | occ | L | focal blurring | NA | n |  |
|  |  |  |  |  |  | fr | bilat (re>li) |  |  |  |  |
| 17 | f | 7 | | 6y | 1 | temp | L | focal signal abnormality | Glioneuronal tumour | y | Ia |
| 18 | m | 2 | | 9m | 1.68 | fr | L | focal blurring | NA | n |  |
| 19 | f | 6 | | 23m | 4.6 | fr | L | malformation | Not resected | n | IV |
|  |  |  |  |  |  | temp | L | focal blurring | FCD IIa | y |  |
| 20 | f | 4.00 | | 2y | 2 | temp | L | focal signal abnormality | FCD IIb | y | Ia |
| 21 | m | 11 | | 4y | 7 | temp (pole inf gyrus) | R | focal blurring | NA | n |  |
| 22 | f | 2 | | 13m | 1 | temp | R | focal signal abnormality | FCD IIb | y | Ia |
| 23 | m | 12 | | 1.5y | 10.50 | temp | R | focal signal abnormality | HS (ILAE 1) | y | IV |
| 24 | m | 13 | | 6y | 7 | fr, insular | R | large focal cortical abnormality | Polymicrogyria | y | Ia |
| 25 | m | 6.00 | | 7m | 5.93 | fr | R | focal blurring | NA | n |  |
| 26 | f | 15 | | 11y | 4 | fr (precentral gyrus) | L | focal signal abnormality | NA | n |  |
| 27 | m | 10.00 | | 2m | 9.98 | insula | L | focal blurring | thermal ablation of left insula | y | Ia |
| 28 | m | 10.00 | | 6y4m | 3.94 | fr (sup, mid gyri) | R | focal signal abnormality | FCD IIb | y | Ia |
| 29 | f | 21.00 | | 8m | 20.92 | fr (lobe, inf sulcus) | R | focal blurring | FCD IIa | y | Ia |
| 30 | m | 7.00 | | 3y | 4.00 | fr | L | transmantle sign | FCD IIb | y | Ia |
| 31 | f | 5.00 | | 6.5m | 4.94 | occ | R | focal blurring | FCD IIb | y | Ia |
| 32 | f | 3 | | 1y | 2 | fr operculum | R | focal signal abnormality | FCD IIb | y | Ia |
| 33 | m | 5.00 | | 1y | 4.00 | temp (inf gyrus) | L | focal signal abnormality | FCD IIb | y | Ia |

**Table 2: Patient demographics**. Age and disease duration are presented in years, while seizures onset is presented in years (y), months (m) or weeks (w). The lesion location is reported according to the MRI-based radiological report. If multiple lesion locations are reported in patients who underwent surgery, all locations were resected. Surgery outcome for the patients who underwent surgery is classified using the Engel post-operative surgical outcome according to Engel classification ^47^, Ia = completely seizure free, III = worthwhile improvement, IV = no worthwhile improvement. Abbreviations: m=male, f=female, L=left, R=right, fr= frontal pole, inf= inferior, occ=occipital pole, par=parietal, post=posterior, sup=superior, temp=temporal lobe, FCD=focal cortical dysplasia, HS=hippocampal sclerosis, n=no, y=yes.

| Patients | FLAIR | MPRAGE | ICVF | ODI | μFA | μAD | μMD | μRD | Diff | μ  Extra-neurite MD | μ  Extra-neurite RD | INVF | FA | MD |
| --- | --- | --- | --- | --- | --- | --- | --- | --- | --- | --- | --- | --- | --- | --- |
| 1 | 3 | 2.5 | 3.75 | 1.5 | 1.5 | 2.5 | 3 | 3.5 | 2 | 1.5 | 2.75 | 3.5 | 1.5 | 1.5 |
| 2 | 2 | 1.5 | 1.5 | 1 | 1 | 1 | 1 | 1 | 1 | 1 | 1 | 1.5 | 1 | 1 |
| 3 | 3.5 | 2 | 3 | 2 | 1.5 | 1.75 | 1.5 | 2.5 | 3 | 1 | 2 | 2.5 | 2 | 2 |
| 4 | 3 | 2 | 1.5 | 1 | 1 | 1 | 1 | 1.5 | 1 | 1 | 1.25 | 1.5 | 1 | 1.5 |
| 5 | 1.5 | 2 | 2 | 1 | 1 | 1 | 1 | 1.5 | 1.5 | 1.5 | 1 | 1.5 | 1 | 1 |
| 6 | 2.5 | 1.5 | 1.5 | 1 | 2 | 1.5 | 1.5 | 1.5 | 1 | 1 | 1 | 1 | 1 | 2 |
| 7 | 3 | 2.5 | 3 | 1.5 | 2 | 2 | 3 | 2 | 1.5 | 3 | 3 | 1.5 | 1.5 | 2.5 |
| 8 | 2.5 | 1.5 | 1.5 | 1 | 1.5 | 1.5 | 1 | 1.5 | 1.5 | 2 | 1 | 1.5 | 1 | 1.5 |
| 9 | 2 | 1.75 | 2 | 1 | 1.5 | 1.75 | 1.5 | 1.5 | 1.5 | 1.5 | 2 | 1 | 1.5 | 1.5 |
| 10 | 3.5 | 3 | 1.5 | 1 | 1.5 | 1 | 1 | 2 | 2.25 | 2 | 1 | 1.5 | 1 | 2 |
| 11 | 4 | 3 | 3.5 | 1 | 1.5 | 2 | 2 | 4 | 1 | 2 | 3.25 | 2.5 | 1.5 | 1.5 |
| 12 | 3.5 | 3.5 | 2.5 | 1 | 1.5 | 1.5 | 1.5 | 1.5 | 2 | 1.5 | 1.5 | 2 | 1 | 1.5 |
| 13 | 4 | 3 | 2.5 | 1.5 | 1.5 | 1 | 1 | 3 | 1 | 1 | 1.5 | 2 | 1 | 2 |
| 14 | 4 | 4 | 4 | 2 | 1 | 3 | 2 | 3 | 3 | 2 | 2 | 3 | 1 | 2 |
| 15 | 4 | 3 | 3 | 1 | 2 | 2 | 1 | 2.5 | 2 | 1.5 | 1.5 | 2 | 1 | 1.5 |
| 16 | 2.5 | 2 | 3 | 1 | 1 | 1 | 1.5 | 2 | 1.5 | 1.5 | 1.5 | 2 | 1.5 | 1.5 |
| 17 | 4 | 4 | 3.5 | 3.25 | 3.25 | 2.5 | 3 | 3.25 | 3 | 3 | 3 | 3.5 | 2.5 | 3 |
| 18 | 3.5 | 3.5 | 2.5 | 2 | 1.5 | 1 | 1.5 | 2 | 1 | 1 | 1.5 | 2 | 1.5 | 2 |
| 19 | 2.5 | 2.5 | 2 | 1.5 | 1.5 | 1.5 | 2.75 | 2.75 | 1.5 | 1.5 | 1.5 | 1.5 | 1 | 1.5 |
| 20 | 3 | 2 | 1 | 1.5 | 1 | 1 | 1.5 | 1 | 1 | 1 | 1 | 1 | 1 | 1 |
| 21 | 2 | 1 | 2 | 1 | 1 | 1 | 1 | 1 | 1 | 1 | 1 | 2 | 1 | 1 |
| 22 | 3.5 | 3.5 | 3.5 | 1 | 1.5 | 2 | 2.5 | 2.5 | 1.5 | 1.5 | 2.5 | 2 | 1.5 | 2 |
| 23 | 3 | 2.5 | 3 | 1 | 1.5 | 1.5 | 2 | 2.5 | 2 | 1.5 | 2 | 2 | 1.5 | 1.5 |
| 24 | 4 | 4 | 4 | 2.5 | 3.5 | 3.5 | 3 | 3.5 | 4 | 3.5 | 2.5 | 4 | 3 | 2.5 |
| 25 | 2 | 2 | 2 | 2 | 1.25 | 1.75 | 2 | 1.5 | 1 | 1 | 1 | 1.5 | 1 | 1 |
| 26 | 3.5 | 4 | 4 | 1.5 | 2 | 3.25 | 3 | 3 | 3.5 | 3 | 3.25 | 3 | 2.5 | 3.25 |
| 27 | 2 | 2 | 3 | 1 | 1 | 2 | 1 | 1 | 1 | 1 | 1 | 2.5 | 1 | 1 |
| 28 | 3.5 | 4 | 4 | 1.5 | 1 | 1.5 | 3 | 2.5 | 1 | 2 | 3.5 | 4 | 1.5 | 3.5 |
| 29 | 2 | 2.5 | 2.5 | 1 | 1 | 2 | 2 | 2 | 1.5 | 2.125 | 1.5 | 1.5 | 1 | 1 |
| 30 | 3.5 | 3.5 | 4 | 2 | 2 | 4 | 4 | 4 | 1 | 2.5 | 3 | 4 | 2 | 3.5 |
| 31 | 2.5 | 2.5 | 2 | 1 | 1.5 | 1 | 1.5 | 1.5 | 2 | 1 | 1.5 | 1.5 | 2 | 1.5 |
| 32 | 4 | 3 | 3 | 1 | 2 | 2 | 1.5 | 3 | 1.5 | 1 | 1 | 2 | 1 | 1.5 |
| 33 | 2.5 | 3 | 3.5 | 1 | 1.5 | 1 | 2 | 1.5 | 1 | 2 | 2 | 2.5 | 1 | 2 |

**Table 3: Mean visual assessment scores**. Two expert paediatric neuro-radiologists evaluated the FCD lesion visibility on FLAIR, MPRAGE, NODDI, microscopic and multi-compartment microscopic SMT, and standard DTI images. Scores: 1=not visible, 2=subtle, 3=clear, 4=very clear. ICVF=intracellular volume fraction, ODI = orientation dispersion index, μFA = μ fractional anisotropy, μAD = μAxial diffusivity, μMD = μ mean diffusivity, μRD = μ radial diffusivity, Diff = intrinsic diffusivity, μExtra-neurite MD= extra-neurite μ mean diffusivity, μExtra-neurite RD= extra-neurite μ radial diffusivity, INVF = intra-neurite volume fraction.

| Maps | Spearman correlation coefficient | |
| --- | --- | --- |
|  | ρ | p-value |
| ICVF | 0.44 | 0.01 |
| ODI | 0.30 | 0.085 |
| μFA | 0.41 | 0.019 |
| μAD | 0.17 | 0.343 |
| μMD | 0.31 | 0.083 |
| μRD | 0.59 | <10^-5^ |
| Diff | 0.18 | 0.309 |
| μExtra-neurite MD | 0.17 | 0.348 |
| μExtra-neurite RD | 0.56 | 0.001 |
| INVF | 0.42 | 0.016 |
| FA | 0.43 | 0.013 |
| MD | 0.31 | <10^-5^ |

**Table 4: Correlation between visual scores and quantitative measure of lesion conspicuity**. The table reports the Spearman correlation coefficient between the average visual scores of two neuro-radiologists, and the lesion asymmetry values for each diffusion map. The asymmetry was estimated as the difference between diffusion values sampled in the lesion and homotopic region at 3mm depth from the pial surface. Significance level was set at p-value<0.05. ICVF=intracellular volume fraction, ODI = orientation dispersion index, μFA = microscopic fractional anisotropy, μAD = microscopic axial diffusivity, μMD = microscopic mean diffusivity, μRD = microscopic radial diffusivity, Diff = intrinsic diffusivity, μExtra-neurite MD= extra-neurite microscopic mean diffusivity, μExtra-neurite RD= extra-neurite radial diffusivity, INVF = intra-neurite volume fraction.
